# Ultra-stretchable hydrogels with hierarchical hydrogen bonds

**DOI:** 10.1038/s41598-020-68678-9

**Published:** 2020-07-16

**Authors:** Yujing You, Jian Yang, Qiang Zheng, Ningkun Wu, Zhongda Lv, Zhiqiang Jiang

**Affiliations:** School of Materials Science and Chemical Engineering, Ningbo University of Technology, 201 Fenghua Road, Jiangbei, Ningbo, 315211 Zhejiang China

**Keywords:** Gels and hydrogels, Polymers

## Abstract

Hydrogels are attractive for applications in intelligent soft materials and flexible electronics. Herein, we report a new hydrogel with a hierarchical hydrogen bond system consisting of (1) weak hydrogen bonds between *N*,*N*-dimethylacrylamides (DMAA) and acrylic acids (AAc) and (2) strong multiple hydrogen bonds between 2-ureido-4[1*H*]-pyrimidinone units. By optimizing the ratios of DMAA and AAc and the balance of weak and strong hydrogen bonds, the hydrogels have unique properties. The transparent hydrogels have tunable Young’s modulus (70–1,250 kPa) and are highly stretchable (up to 4,340% strain), tough (fracture energies of 10.8 kJ/m^2^, matching natural rubber) and insensitive to notches when it is highly stretched (λ = 19.6).

## Introduction

Stretchable hydrogels are promising materials for diverse applications, such as biomaterials^1^, force sensors^2,3^, supercapacitors^4^, actuators^5^, optical fibers^6^, stretchable conductors^7^ and soft electronics^8^. Since the hydrogels undergo dynamic actions in these applications, such as stretching, compression and torsion, they should have appropriate mechanical strength, flexibility and stretchability under deformation. Intensive efforts have been devoted to developing tough and elastic hydrogels. Gong et al.^9^ reported a double network hydrogel (DN hydrogel) by introducing a soft hydrogel network with slippery chains into a rigid hydrogel network. The internal fracture of covalent bonds in the brittle network, which dissipates significant energy^10^. The soft network absorbs the energy and prevents the formation of macroscopic crack. DN hydrogels show robust mechanical strength and toughness. However, permanent chemical fracture in DN hydrogels led to poor fatigue resistance.

The toughness and elasticity of DN hydrogel originate from the mechanism of disruption of sacrificial bonds, which dissipated energy^11^. Attempts to introduce other chemical structures or physical crosslinks as sacrificial bonds have been made, such as ionic bonds^12,13^, triblock copolymers^14^ and polyampholytes^15^. Hybrid hydrogel systems by introducing crystallites^16^ and microspheres^17^, micelles^18^, nanocomposite^19,20^ into the hydrogels have also been reported. These reversible crosslinked sacrificial bonds or microstructures synergically dissipated energy, which endows hydrogels with high stretchability and toughness.

Hydrogen bonds are promising dynamic bonds to impart fascinating properties, such as self-healing^21–23^, toughness^24,25^, and shape memory^26^. Hydrogen bonds are stable in the presence of ions and they are different from other aggregates like micelles, inorganic fillers, which have sizes at the nano or micron level and impede the optical properties. Hydrogel base on hydrogen bonds can be transparent, which is favorable to sensors and electronics. However, hydrogels with only one energy-dissipating mechanism by H-bonds cannot achieve satisfactory overall mechanical properties. They are either soft and tough or strong and brittle^27–29^.

Here, we report a new multiple energy-dissipating hydrogel with hierarchical hydrogen bonds. The hydrogel was synthesized by copolymerization of acrylamide, acrylic acid and a 2-ureido-4[1*H*]-pyrimidinone motif (UPy). 2-Ureido-4[1*H*]-pyrimidinone motif forms quadruple hydrogen bonds and has a high dimerization constant (K_dim_ ≈ 6 * 10^8^ M^−1^ in toluene)^30^, which makes it very attractive building blocks for tough hydrogels. The hydrogel was formed on weak hydrogen bonds between acrylamides and acrylic acids and strong multiple hydrogen bonds between UPy units. The physical dissociation and re-association of hydrogen bonds at different force level dissipates energy and synergically contributes to the hydrogel with remarkable mechanical properties.

## Results and discussion

**Scheme 1:**
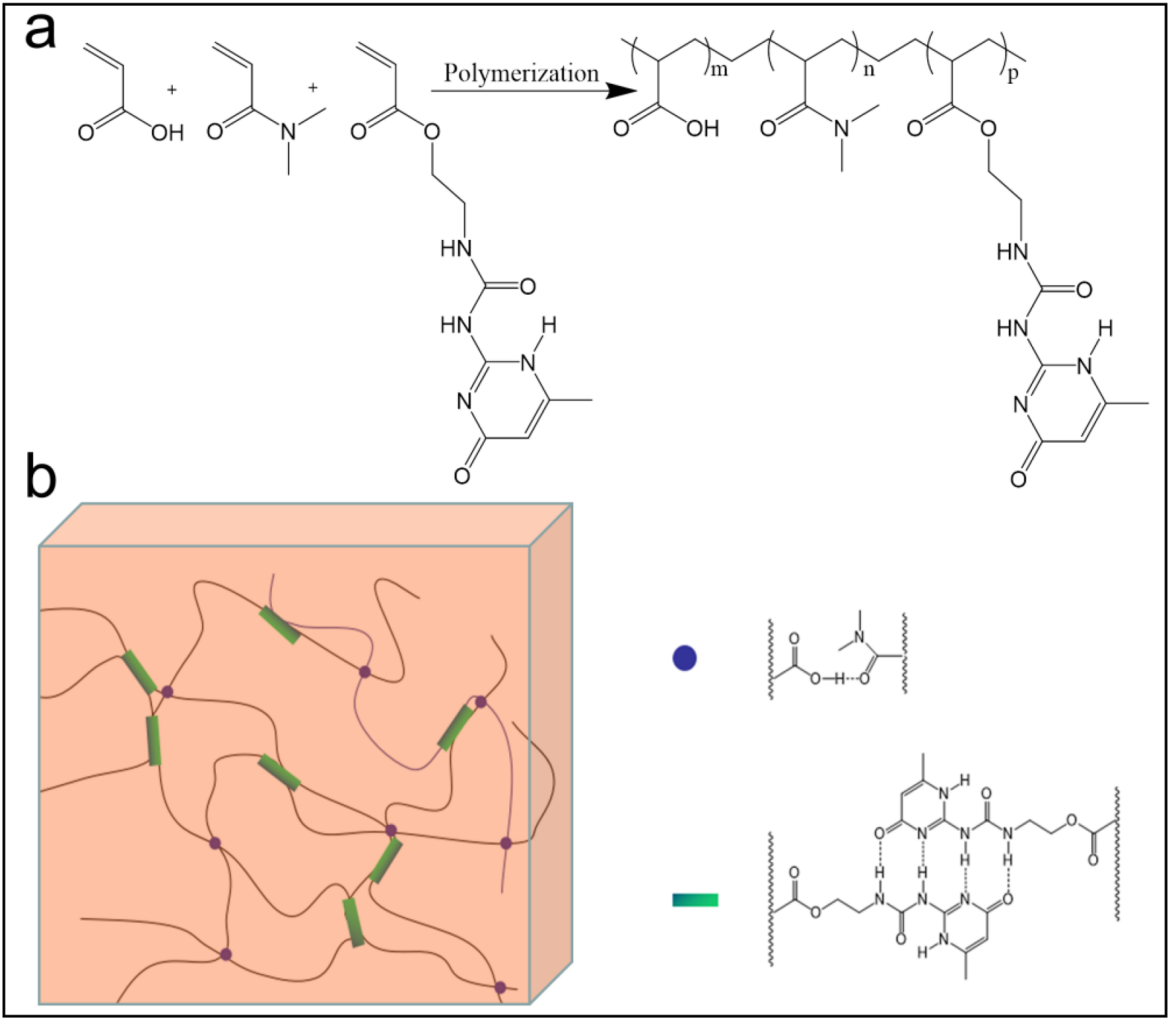
(**a**) Synthesis procedure and the chemical structures of the hydrogel. (**b**) Demonstration of the hierarchical hydrogen bonds in the hydrogel with weak H-bonds between acrylamides and acrylic acids and strong multiple H-bonds between UPy units.

Hydrogels were synthesized by one-pot free radical copolymerization of *N*,*N*-dimethylacrylamide, acrylic acid and 2-(3-(6-methyl-4-oxo-1,4-dihydropyrimidin-2-yl) ureido) ethyl acrylate (UPyEA) under UV radiation (λ = 365 nm) (Scheme 1a). The dimethyl amide group is a strong hydrogen-bond acceptor, while acrylic acid is a potent hydrogen-bond donor. The amide groups of DMAA are able to form hydrogen bonds with the carboxylic acid groups (Scheme 1b). UPyEA was synthesized by coupling 2-isocyanatoethylacrylate with 2-amino-4-hydroxy-6-methyl pyrimidine. The UPy units form self-complementary dimers with quadruple H-bonds in aqueous solutions. Surfactant Triton X-100 was added to the solution to enhance the solubility of UPyEA. UPyEA in the polymer backbone leads to multiple intermolecular hydrogen bonding. The ratios of DMAA and AAc, the concentrations of UPyEA and the pH values of the reactant solutions were modulated. The resulting hydrogels contained 60% water and were transparent and in good shape.

The formation of hydrogen bonding and other molecular interactions affects the position of the involved peaks by shifting to a lower wave number. The absorbance at 1,610 cm^−1^ was attributed to C=O group of the acrylamide unit. The wave number of the C=O peak decreased from 1,610 to 1,590 cm^−1^. This result indicated that there was an interaction between the carboxylic groups of AAc and the amide groups of DMAA. (Figure S2). Hydrogen-bonding energy between amide acceptor and hydroxyl donors is − 4.51 kcal/mol^31^. According to the relationship between hydrogen-bonding energies and hydrogen-bonding equilibrium constants, the log k = 1.66. thus K = 39.8 M^−1^. The hydrogen-bonding constant of UPy (K_dim_ ≈ 6*10^8^ M^−1^) is much bigger than that between DMAA and AAc units. UPy dimers are more rigid and the pairs of DMAA and AAc are loose and dynamical.

**Figure 1:**
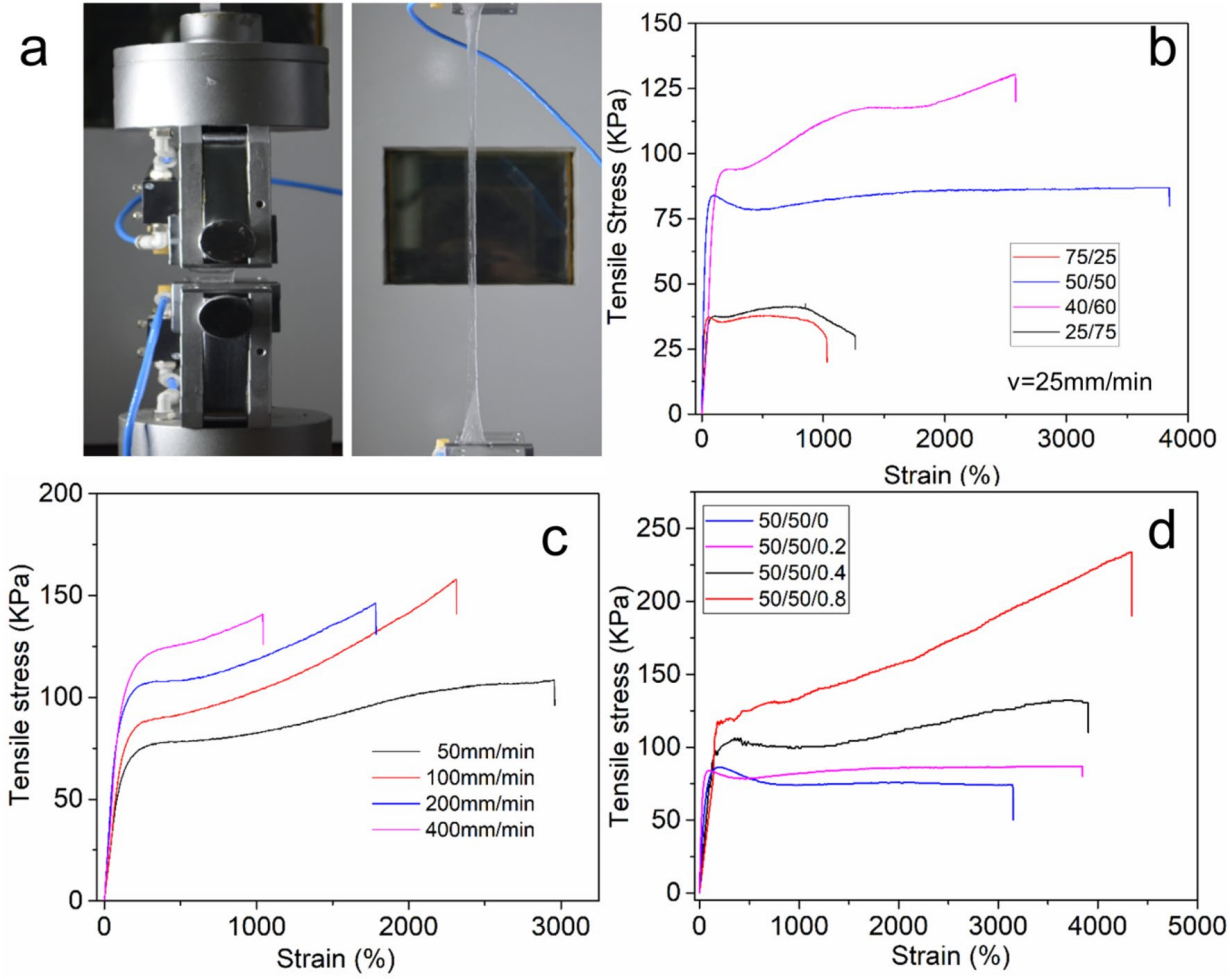
Mechanical properties of the hydrogels. (**a**) Photographs of a hydrogel before and after stretching (v = 25 mm/min). Edit by WPS Office with the version 11.1.0.9513. https://pc.wps.cn/ (**b**) Tensile stress curves of the hydrogels prepared with different DMAA to AAc molar ratios. (**c**) Stress–strain curves of hydrogel D50A50U0.2 stretched at different speeds (v = 50, 100, 200, 400 mm/min). (**d**) Stress–strain curves of the hydrogel (R_DMAA/AAc_ = 50/50) prepared with different UPy molar ratios (c = 0, 0.2%, 0.4%, 0.8%).

The hydrogels were cut into rectangle shapes for tensile tests (Fig. 1a). Figure 1b shows the tensile stress–strain curves of hydrogel with different molar ratios of DMAA and AAc with a stretching speed of 25 mm/min. By tuning the ratio of DMAA and AAc, the density of hydrogen bonding varied which affected the mechanical properties of the hydrogels. Hydrogels synthesized from DMAA or AAc alone are mechanically weak and fragile, however, their copolymer hydrogels are tough and stretchable. Although DMAA units could form hydrogen bonds with each other, in this series of hydrogels their hydrogen bonding with AAc is dominant. The polymer mass concentrations are maintained identical in all the hydrogels, so if the molar ratio of DMAA/AAc deviates from the balanced ratio R_DMAA/AAc_ = 1, the hydrogen density decreased and the network became weak. As shown in Table 1, the modulus of the hydrogel increased from 76 to 283 KPa with the DMAA fraction increasing from 25 to 50%, then decreased to 136 KPa (75%). The tensile strain has the same trends. The hydrogel with the molar ratio of DMAA/AAc/UPyEA = 50/50/0.2 (Denoted as D50A50U0.2) has a big tensile strain of 3,850%.

**Table 1:** Physical properties of various hydrogels.

No.	Molar ratio DMAA/AAc/UPyEA	Modulus (kPa)	Max stress (kPa)	Strain at break (%)	Strain energy density (kJ m^−3^)
1	50/50/0	74 ± 10	74 ± 5	3,150 ± 240	2,370 ± 140
2	50/50/0.2	283 ± 18	87 ± 11	3,850 ± 230	3,230 ± 370
3	50/50/0.4	409 ± 36	132 ± 17	3,900 ± 280	4,330 ± 320
4	50/50/0.8	1,252 ± 108	234 ± 16	4,340 ± 320	7,160 ± 670
5	75/25/0.2	136 ± 11	38 ± 5	1,030 ± 80	370 ± 60
6	40/60/0.2	183 ± 10	130 ± 8	2,580 ± 180	2,840 ± 280
7	25/75/0.2	76 ± 8	41 ± 6	1,260 ± 170	470 ± 80

The hydrogel with the molar ratio of DMAA/AAc = 50/50 has a larger number of hydrogen bonds which resulted in higher crosslinking density. Tensile tests were conducted at other stretching speeds (Fig. 1c). The hydrogen bonds between DMAA and AAc and those between UPy units have a wide strength distribution, which gives dynamic crosslinking of a wide lifetime scale. At high-strain-rate, the hydrogel became harder and less stretchable. By changing the tensile speed from 50 to 400 mm/min, the hydrogel modulus was increased from 693 to 1,126 kPa, but the strain at failure was decreased from 2,960 to 1,040%. The speed of 25 mm/min was chosen for the following tensile tests.

The hydrogels containing more UPy units have higher modulus and stress and bigger tensile strains (Fig. 1d), the control sample D50A50U0 without UPy units displayed Young's modulus of 74 ± 10 kPa, while the D50A50U0.2 hydrogel had Young's modulus of 283 ± 18 kPa. It is close to the characteristic modulus values of human tissues and organs. Many native tissues have modulus in this range, e.g. human nasal cartilage (≈ 250–440 kPa)^32^ and human skin (≈200–250 kPa)^33^. Hydrogel containing 0.4% UPy units showed an increase to 409 ± 36 kPa. Hydrogels with 0.8% UPy units exhibited ≈ 1,700% increase in elastic modulus (1,252 ± 108 kPa) as compared with the control sample D50A50U0 without UPy. It also has the biggest tensile strain of 4,340 ± 320%. These hydrogels dissipated energy effectively, as shown by pronounced hysteresis. The area under stress–strain curves of a hydrogel gives the hydrogel toughness.

Hydrogel without UPy units has Young's modulus of 74 ± 10 kPa, while the hydrogels with 0.2%, 0.4%, 0.8% UPy units had Young's modulus of 283 ± 18 kPa, 409 ± 36 kPa, and 1,252 ± 108 kPa respectively. The tensile strain also increased from 3,850% (D50A50U0.2) to 4,340% (D50A50U0.8). For thermoset elastomers like rubber, the increase of modulus at higher cross-linker levels trades off with the maximum elongation. Thus higher cross-linking density results in more rigid and less elastic rubbers^34^. In contrast, in our hydrogel samples, the modulus and tensile strength increased consistently without sacrificing the extensibility. This can be attributed to the increased energy dissipation ability of the hydrogen bonds. While the modulus is increased at higher cross-linking density, the reversibly unfolding modules can act as energy dissipating units to prevent fracture formation. The hydrogen bonds between DMAA and AAc are reversible crosslinking that break and reform under stress. UPy units in the hydrogel form self-complementary dimers near other units on neighboring chains. When force was applied to the hydrogel, the interchain UPy dimers disassociate, which contributes to the hydrogels’ super stretchablility.

**Figure 2:**
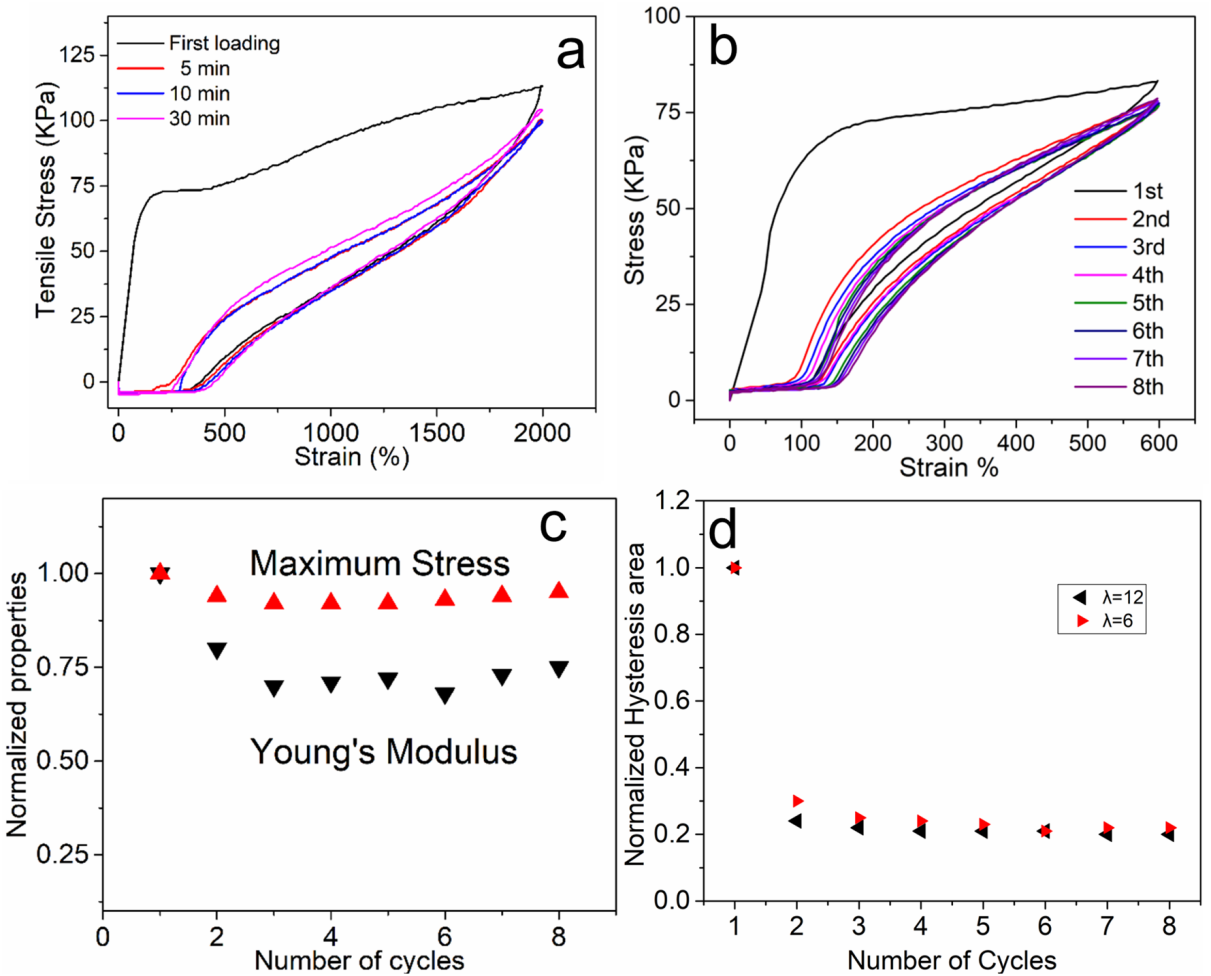
(**a**) Stress–strain curves of the hydrogel D50A50U0.2 cyclic stress–strain tests (up to 2,000% strain) as prepared (black), stretched 5 min (red), 10 min (blue), 30 min (pink) after different waiting times between two successive measurements. (**b**) Stress–strain curves of the hydrogel D50A50U0.2 in cyclic stress–strain tests without any rest time between the cycles. (**c**) Calculated decay of maximum stress and Young’s modulus from stress–strain curves of the hydrogel in 8 cyclic tensile tests. (**d**) Normalized hysteresis ratios for the hydrogels stretched to λ = 6 and 12 in the cyclic tests.

The recovery of hydrogel deformation was studied by cyclic tensile tests (Fig. 2). The hydrogel specimen was first extended to a strain of 2,000% at room temperature and then unloaded at the same speed (v = 25 mm/min). A certain waiting time was added between the cycles. As shown in Fig. 2a, the first loading–unloading cycle shows a notable hysteresis area with a residual strain (390%), indicating the plastic deformation. Longer rest time contributes to the slightly better recovery, which means that a bigger time scale is beneficial to the reformation of the sacrificial hydrogen bonds in the hydrogel. D50A50U0.2 hydrogel sample was subjected to eight consecutive loading–unloading cycles without any rest time between the cycles (Fig. 2b).

As shown in Fig. 2c, the most significant drop in Young’s modulus and maximum stress is observed after the first cycle. In the following cycles, the modulus and stress decreased much slowly. Tensile force may induce the rapture of weak hydrogen bonds and the entangled polymer chains rearranged. The hydrogen bonds re-associated with neighboring groups which maintain the hydrogel's shape integrity as it changed into a bigger dimension in the tensile direction. The hydrogel displayed similar Young's modulus and maximum stress in the following cycles because hydrogen bonds are reversible physical bonds and there is no permanent chemical structural damage in the hydrogel. Figure 2d presents the hysteresis ratios for two stretch values (λ = 6, 12). Despite the large strain variation in stretch, similar normalized hysteresis areas changes were observed for these two cyclic tests, which suggested the same kind of microscopic structural changes.

**Figure 3:**
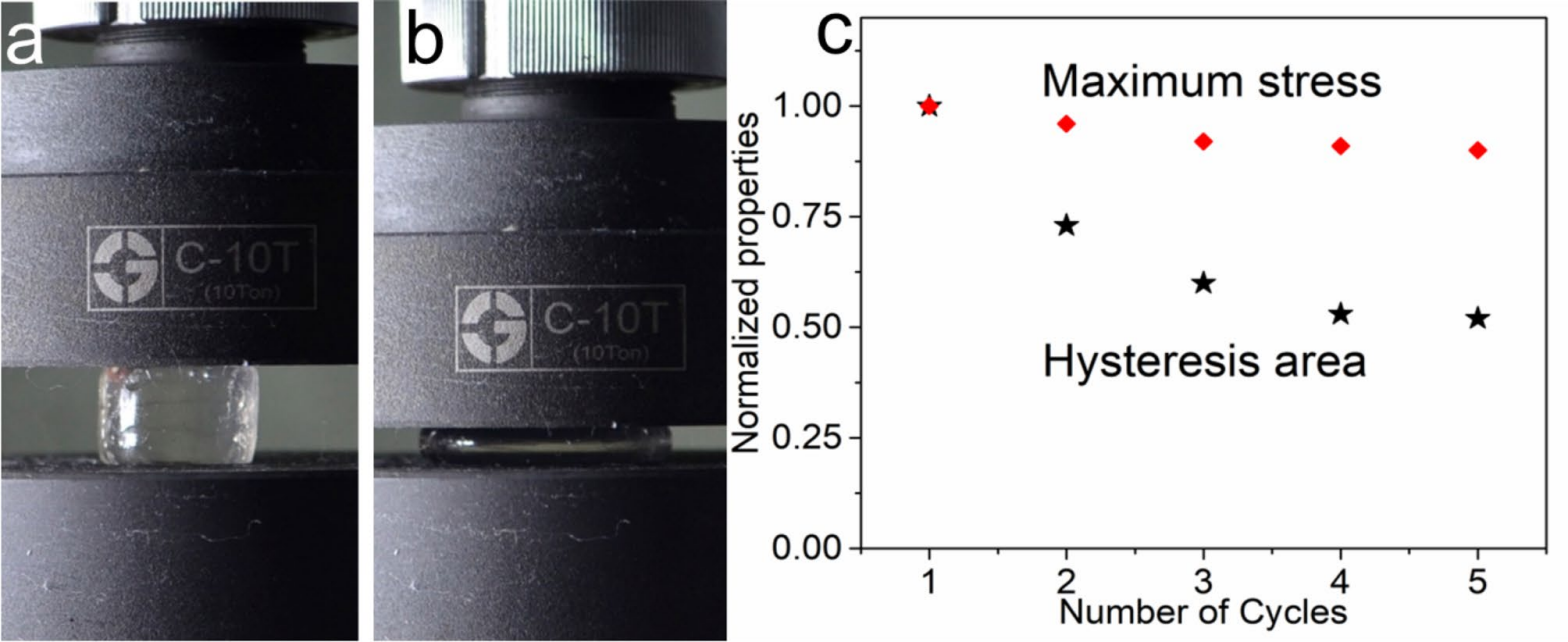
| Photographs of D50A50U0.2 hydrogel before (**a**) and after compression. (**b**) (v = 5 mm/min). (**c**) Calculated decay of maximum stress and hysteresis area from stress–strain curves of the hydrogel in 5 cyclic compression tests. (**a**, **b**) was edited by WPS Office with the version 11.1.0.9513. https://pc.wps.cn/.

We also conducted cyclic compression tests in an attempt to further investigate the properties of the hydrogel (Fig. 3a, b). The hydrogel maintained its shape integrity under large strain (80%). After 5 compression cycles, the maximum stress decreased slightly (only 10%). The sample shows a notable residual hysteresis area after the first unloading. However, it decayed much slowly in the following compression (Fig. 3c). The relaxation force is small and the UPy dimers is relatively rigid, thus, the hydrogels showed residual strain after the cyclic compression. Stress-relaxation measurements were carried out to further investigate the viscoelastic behavior of the hydrogels (see Figure S3). The ratios of DMAA and AAc were maintained at 50/50, the content of UPy units increased gradually to 0.8 mol%. The hydrogels without UPy appeared elastic. The hysteresis ratio after 8 cycles was 0.97, suggesting nearly full recovery. Hydrogen-bonds between DMAA and AAc are dynamic and allow for recurring dissociation and reformation until the network is fully relaxed. With the incorporation of UPy units, the hysteresis areas increased and the hysteresis ratio decreased to 0.76 for UPy = 0.4 and 0.65 for UPy = 0.8. The dissociation of hydrogen bonds between UPy units allows for significant energy dissipation during compression. However, the hydrogen-bonds between UPy units appeared rigid and the hydrogels did not get full recovery of its original structure during relaxation.

**Figure 4:**
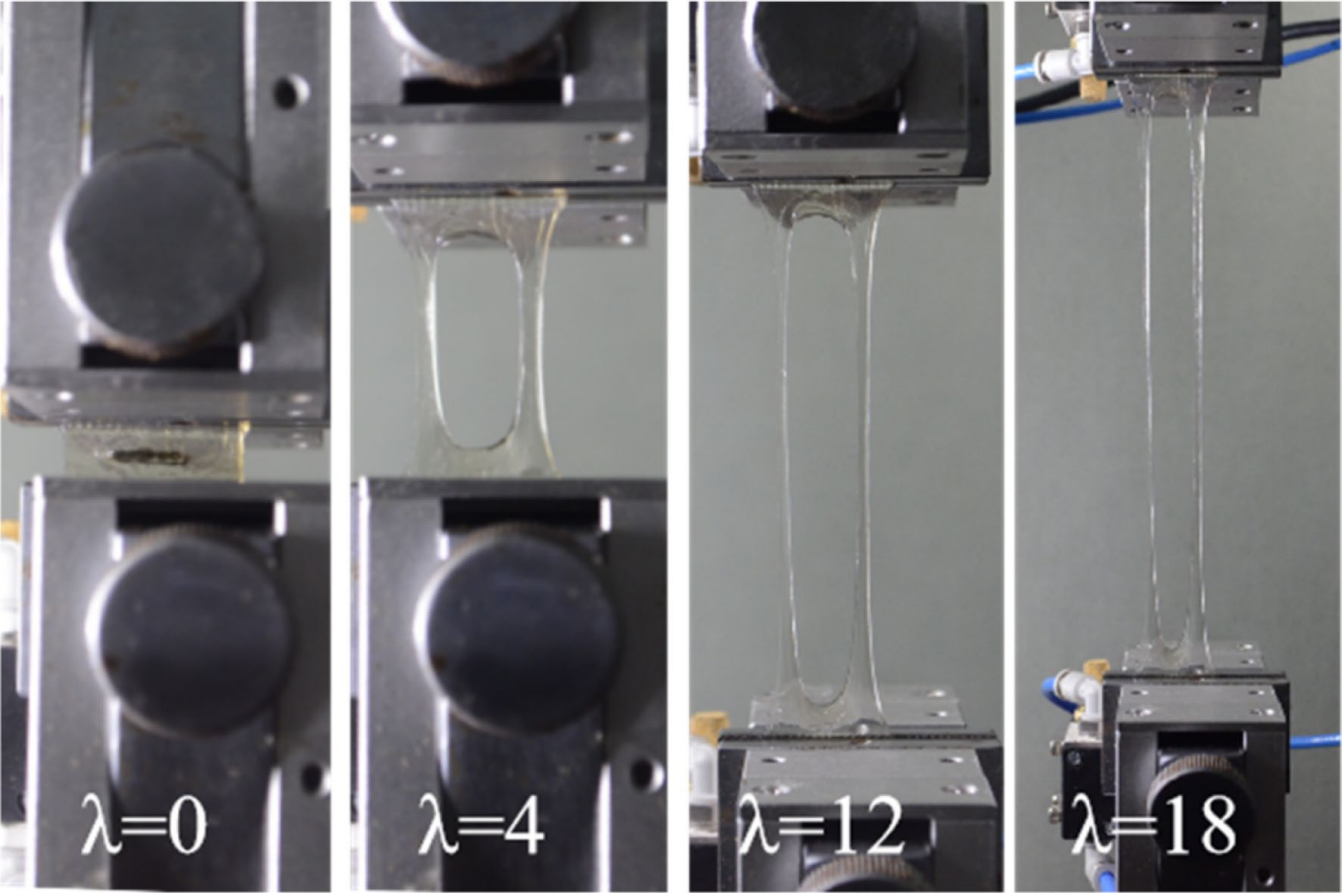
Photographs of D50A50U0.2 hydrogel with a notch in tensile test (v = 25 mm/min). The hydrogel (width = 24 mm, length = 15 mm) containing the notch (width = 12 mm) was stretched to 19.6 times its initial length. Edited by WPS Office with the version 11.1.0.9513. https://pc.wps.cn/.

Most hydrogels are sensitive to notches and rupture if they contain a small crack. By introducing hierarchical hydrogen bonds into the polymer network, our hydrogel displayed excellent notch-insensitive property. A crack of 12 mm in length was made in D50A50U0.2 hydrogel specimen (w = 24 mm), then the hydrogel was extended to an ultimate stretch of λ = 19.6 (Fig. 4). The notch remained extremely stable. The hydrogel has fracture energies of 10.8 KJm^−2^, which is bigger than cartilage^35^ (ca. 1,000 Jm^−2^) and comparable to natural rubbers^36^ (ca. 10,000 Jm^−2^). The notch sensitivity is related to the hydrogel’s ability to dissipate energy at the apex of the crack before they propagate into macroscopic cracks. When the hydrogel is stretched, hydrogen bonds across the plane of the crack break and reform, which elicit hysteresis in the bulk of the network and contribute to the toughness^37^.

## Conclusions

In summary, we have designed hydrogels with a hierarchical H-bond system, which is composed of weak hydrogen bonds between DMAA and AAc and strong multiple hydrogen bonds between UPy units. The hierarchical energy-dissipating structure synergically contributes to the mechanical properties of the hydrogels, such as excellent toughness and ultra-stretchability. The hydrogel is highly compressible and is insensitive to notches when it is highly stretched with the fracture energy close to natural rubbers. Notably, the mechanical properties such as modulus, toughness and stretchability can be easily controlled by simply modulating the chemical compositions. The ultra-stretchable hydrogels with these desirable properties may expand their potential applications in soft ionotronics, sensors and actuators.

## Experimental section

### Materials

*N*,*N*-dimethylacrylamide, acrylic acid, 2-isocyanatoethylacrylate, 2-amino-4-hydroxy-6-methyl pyrimidine, and photoinitiator α-ketoglutaric acid were purchased from Sigma Aldrich and used without further purification.

### Hydrogel synthesis

The hydrogel was synthesized by free radical polymerization of DMAA, AAc and UPyEA in aqueous solution. Typically, DMAA (9.26 g), AAc (6.74 g), UPyEA (0.20 g), α-ketoglutaric acid (photoinitiator, 0.1 g) and Triton X-100 (surfactant, 0.1 g) were added into a 50 mL plastic vial. Distilled water was added and the final volume of the solution was carefully adjusted to be 40 mL. The monomers were mixed by vortex and dissolved by ultra-sonification for 10 min until a clear solution was obtained. Then it was purged with nitrogen for 30 min. The reactant solution was cast into a plastic box (12 × 8 × 2 cm^3^) and irradiated by a UV lamp (365 nm, 20 W) for 30 min until the hydrogel was formed. The hydrogel with the box was put into a plastic bag (24 × 15 cm^2^), sealed, and put into an oven (50 °C). After 8 h of reaction, the hydrogel was carefully taken out for tests.

### Matrix mechanical properties measurement

Rectangular specimens (30 × 15 × 1.5 mm^3^) coated with silicone oil were stretched by an Instron machine (model 3,342 with a load cell of maximum 2000 N). For notched samples, an edge crack of length 6 mm was cut using a razor blade in the middle of the gauge section of the specimen. The stretch rate was fixed at 25 mm min^−1^. From the stress-stretch curves of the unnotched and notched specimens, the fracture energies were calculated according to the literature^20^. The compressive measurements of the hydrogels were conducted with a load cell of a maximum 5000 N was used. The hydrogel samples were cut into cylinders (22.5 mm in diameter and 28 mm in height). The crosshead speed is set at 5 mm/min.

### Data analysis

The data were described as means and standard deviations for 5 samples. The mean value was statistically compared among the groups using two-way analysis of variance (ANOVA) with the Tukey’s honest significant difference test for posthoc multiple comparisons. A value of *p* < 0.05 was considered statistically significant.

## Supplementary information

Supplementary file1.

## References

[1] Rahimi R (2018). Laser-enabled processing of stretchable electronics on a hydrolytically degradable hydrogel. Adv. Healthc. Mater..

[2] Liu H (2018). Biofriendly, stretchable, and reusable hydrogel electronics as wearable force sensors. Small.

[3] Ge G (2018). Stretchable, transparent, and self-patterned hydrogel-based pressure sensor for human motions detection. Adv. Func. Mater..

[4] Guo Y, Zheng K, Wan P (2018). A flexible stretchable hydrogel electrolyte for healable all-in-one configured supercapacitors. Small.

[5] Dong L, Agarwal AK, Beebe DJ, Jiang H (2006). Adaptive liquid microlenses activated by stimuli-responsive hydrogels. Nature.

[6] Wang L (2018). Ultrasoft and highly stretchable hydrogel optical fibers for in vivo optogenetic modulations. Adv. Opt. Mater..

[7] Lin S (2016). Stretchable hydrogel electronics and devices. Adv. Mater..

[8] Keplinger C (2013). Stretchable, transparent, ionic conductors. Science.

[9] Gong JP, Katsuyama Y, Kurokawa T, Osada Y (2003). Double-network hydrogels with extremely high mechanical strength. Adv. Mater..

[10] Gong JP (2010). Why are double network hydrogels so tough?. Soft Matter.

[11] Kolmakov GV, Matyjaszewski K, Balazs AC (2009). Harnessing labile bonds between nanogel particles to create self-healing materials. ACS Nano.

[12] Yang Y, Wang X, Yang F, Wang L, Wu D (2018). Highly elastic and ultratough hybrid ionic-covalent hydrogels with tunable structures and mechanics. Adv. Mater..

[13] Lin P, Ma S, Wang X, Zhou F (2015). Molecularly engineered dual-crosslinked hydrogel with ultrahigh mechanical strength, toughness, and good self-recovery. Adv. Mater..

[14] Zhang HJ (2016). Tough physical double-network hydrogels based on amphiphilic triblock copolymers. Adv. Mater..

[15] Sun TL (2013). Physical hydrogels composed of polyampholytes demonstrate high toughness and viscoelasticity. Nat. Mater..

[16] Li J, Suo Z, Vlassak JJ (2014). Stiff, strong, and tough hydrogels with good chemical stability. J. Mater. Chem. B.

[17] Duan J, Liang X, Guo J, Zhu K, Zhang L (2016). Ultra-stretchable and force-sensitive hydrogels reinforced with chitosan microspheres embedded in polymer networks. Adv. Mater..

[18] Hu X, Vatankhah-Varnoosfaderani M, Zhou J, Li Q, Sheiko SS (2015). Weak hydrogen bonding enables hard, strong, tough, and elastic hydrogels. Adv. Mater..

[19] Hu Z, Chen G (2014). Novel nanocomposite hydrogels consisting of layered double hydroxide with ultrahigh tensibility and hierarchical porous structure at low inorganic content. Adv. Mater..

[20] Sun J-Y (2012). Highly stretchable and tough hydrogels. Nature.

[21] Taylor DL (2016). M. in het Panhuis, self-healing hydrogels. Advanced Materials.

[22] Fan H, Wang J, Jin Z (2018). Tough, swelling-resistant, self-healing, and adhesive dual-cross-linked hydrogels based on polymer-tannic acid multiple hydrogen bonds. Macromolecules.

[23] Chen S (2016). Poly(sebacoyl diglyceride) cross-linked by dynamic hydrogen bonds: a self-healing and functionalizable thermoplastic bioelastomer. ACS Appl. Mater. Interfaces..

[24] Beijer FH, Kooijman H, Spek AL, Sijbesma RP, Meijer EW (1998). Self-complementarity achieved through quadruple hydrogen bonding. Angew. Chem. Int. Ed..

[25] Wang YJ (2019). Ultrastiff and Tough supramolecular hydrogels with a dense and robust hydrogen bond network. Chem. Mater..

[26] Jiang Z-C (2017). Shape memory polymers based on supramolecular interactions. ACS Appl. Mater. Interfaces..

[27] Yang J, Gong C, Shi F-K, Xie X-M (2012). High strength of physical hydrogels based on poly(acrylic acid)-g-poly(ethylene glycol) methyl ether: role of chain architecture on hydrogel properties. J. Phys. Chem. B.

[28] Song G (2013). Facile fabrication of tough hydrogels physically cross-linked by strong cooperative hydrogen bonding. Macromolecules.

[29] Dai X (2015). A mechanically strong, highly stable, thermoplastic, and self-healable supramolecular polymer hydrogel. Adv. Mater..

[30] Söntjens SHM, Sijbesma RP, van Genderen MHP, Meijer EW (2000). Stability and lifetime of quadruply hydrogen bonded 2-ureido-4[1*H*]-pyrimidinone dimers. J. Am. Chem. Soc..

[31] Hao M-H (2006). Theoretical calculation of hydrogen-bonding strength for drug molecules. J. Chem. Theory Comput..

[32] Griffin MF, Premakumar Y, Seifalian AM, Szarko M, Butler PEM (2015). Biomechanical characterisation of the human nasal cartilages; implications for tissue engineering. J. Mater. Sci. Mater. Med..

[33] Li C, Guan G, Reif R, Huang Z, Wang RK (2012). Determining elastic properties of skin by measuring surface waves from an impulse mechanical stimulus using phase-sensitive optical coherence tomography. J. R. Soc. Interface.

[34] James EMB (1994). Erman.

[35] Simha NK, Carlson CS, Lewis JL (2004). Evaluation of fracture toughness of cartilage by micropenetration. J. Mater. Sci. Mater. Med..

[36] Lake GJ (1995). Fatigue and fracture of elastomers. Rubber Chem. Technol..

[37] Kushner AM, Gabuchian V, Johnson EG, Guan Z (2007). Biomimetic design of reversibly unfolding cross-linker to enhance mechanical properties of 3D network polymers. J. Am. Chem. Soc..

