# Supplementary material for: Ultra-stretchable hydrogels with hierarchical hydrogen bonds
Source: Sci Rep. 2020 Jul 16;10:11727. doi: 10.1038/s41598-020-68678-9 (PMC7366625; doi:10.1038/s41598-020-68678-9)
Supplement: Supplementary file 1 — Supplementary file1. [file 41598_2020_68678_MOESM1_ESM.docx]

Supporting Information

**Ultra-stretchable hydrogel with hierarchical hydrogen bonds**

Yujing You, Jian Yang, Qiang Zheng, Ningkun Wu, Zhongda Lv and Zhiqiang Jiang*

School of Materials Science and Chemical Engineering, Ningbo University of Technology. 201 Fenghua Road, Jiangbei, Ningbo, Zhejiang, 315211, China.

**
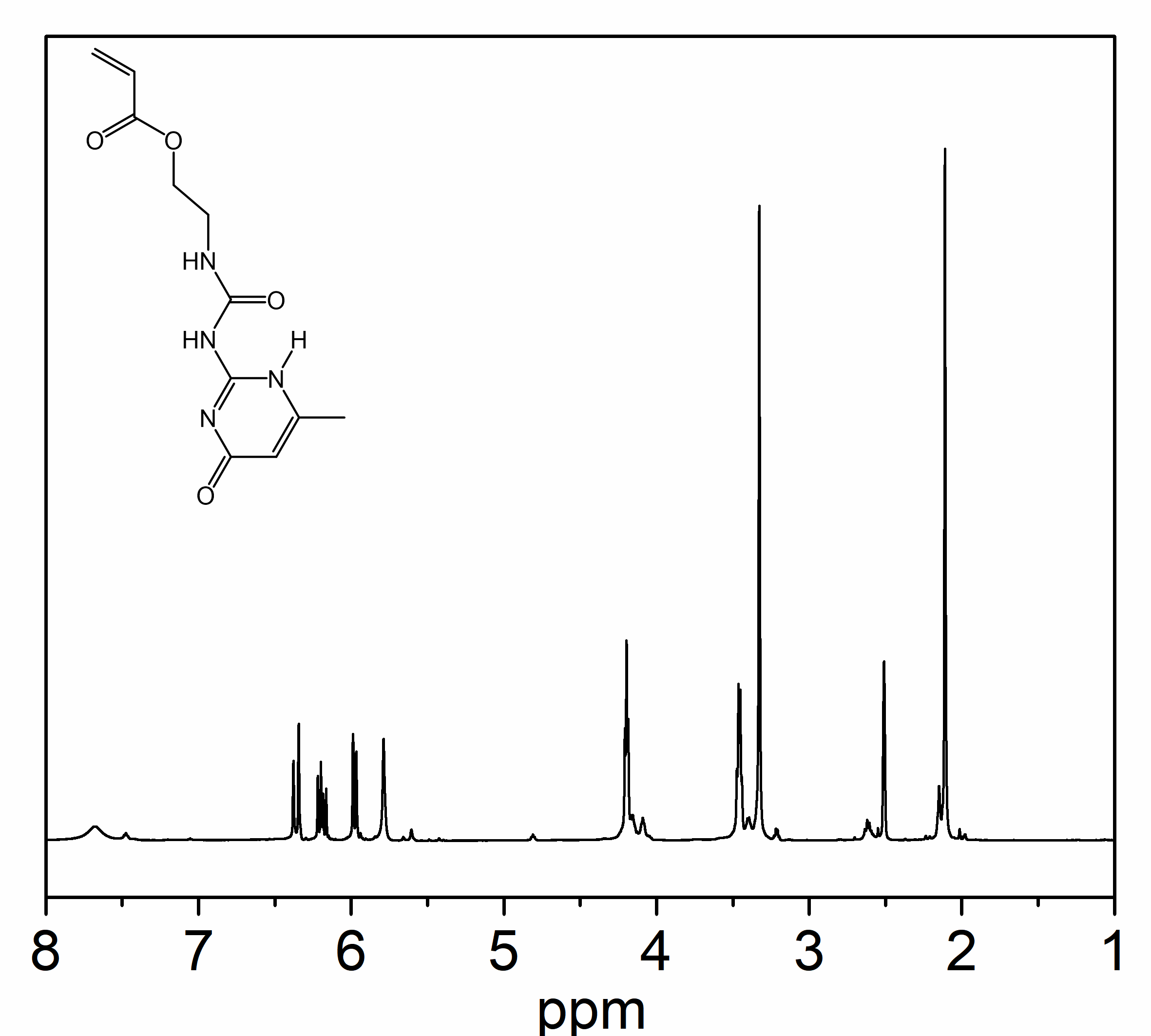
**

**Figure S1.** NMR of UPyEA

**
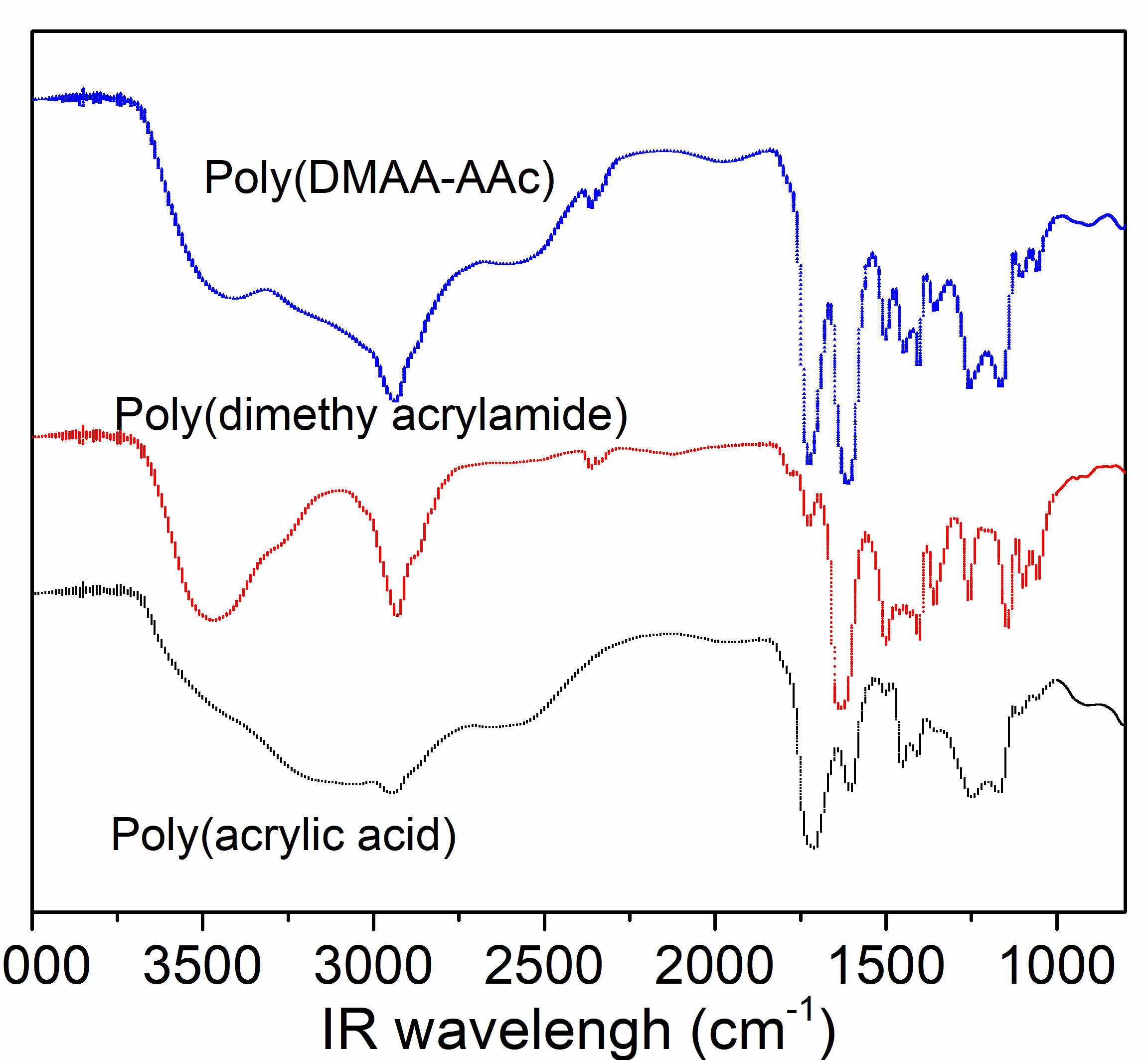
**

**Figure S2. IR spectrum of poly(acrylic acid), poly(dimethy acryamide) and poly(DMAA-AAc)**


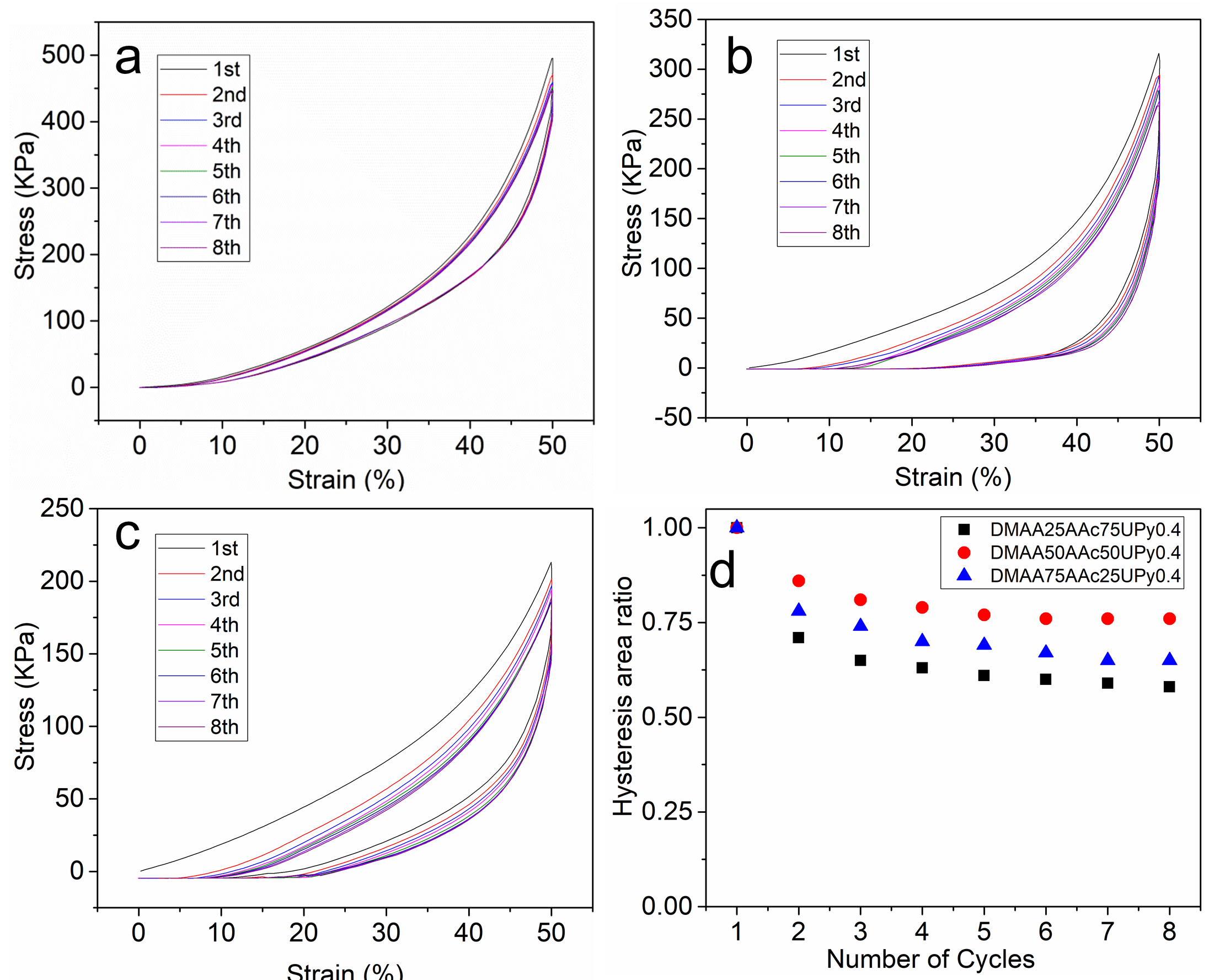


**Figure S3** | a. Stress-strain curves of the hydrogels with various DMAA/AAc molar ratios (a, D25A75U0.4, b, D50A50U0.4 and c. D75A25U0.4) cyclic compression tests (up to 50% strain). d. Normalized hysteresis ratios for the hydrogels in the cyclic tests.

The ratios of DMAA and AAc were maintained at 50/50, the content of UPy moieties increased gradually to 0.8 mol %. The gels without UPy appeaed elastic. The hysteresis ratio after 8 cycles was 0.97, suggesting nearly full recovery. Hydrogen-bonds between DMAA and AAc are weaker and allow for recurring dissociation and reformation until the network is fully relaxed. With the incorporation of UPy moieties, the hysteresis areas increased during the cycles, and hysteresis ratio decreased to 0.76 for UPy=0.4 and 0.65 for UPy=0.8. The dissociation of hydrogen bonds between UPy moieties allows for significant energy dissipation during compression. However, the Hydrogen-bonds between UPy moieties appeared rigid and the gels did not get full recovery of its original structure during relaxation.


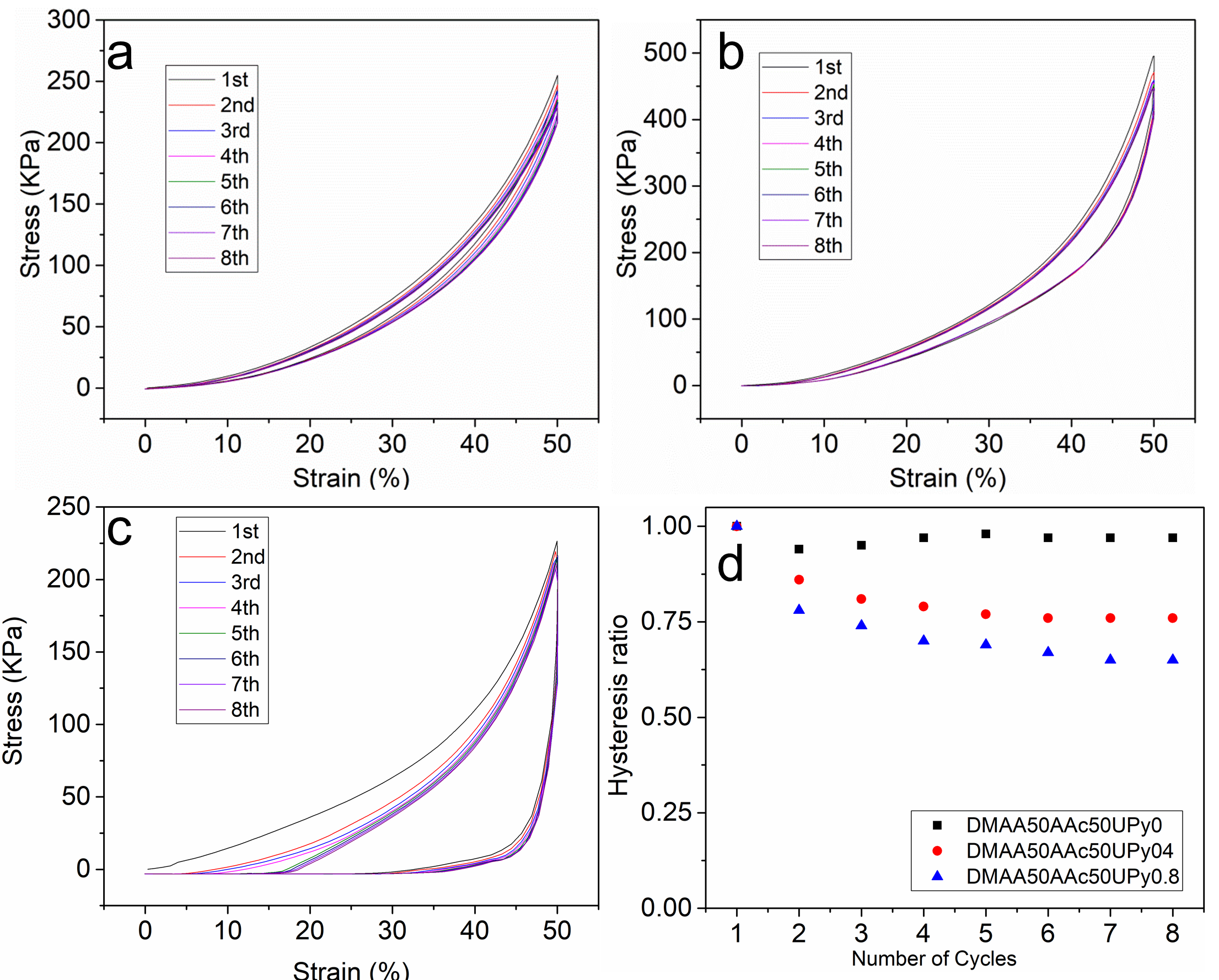


**Figure S4** | a. Stress-strain curves of the hydrogels with changing UPy units molar contents. (a, D50A50U0, b, D50A50U0.4 and c. D50A505U0.8) cyclic compression tests (up to 50% strain). d. Normalized hysteresis ratios for the hydrogels in the cyclic tests.

The gels with different UPy units concentration were compressed with a strain limit of 50% without relaxation time between the intervals. As shown in Figure 5a, After the second cycle of the gel with DMAA/AAc= 50/50, it showed hysteresis (16.58 KJ.m^-3^, hysteresis ratio 0.83) and the residual strain is 2.3%. However, for the hydrogel DMAA/AAc/UPy=25/75/0.4, the first loading-unloading cycle showed a large hysteresis area (23.06 KJ.m^-3^, hysteresis ratio 0.71) with a notable residual strain (21.4%). Hydrogel DMAA/AAc/UPy=75/25/0.4 also showed a hysteresis area (33.03 KJ.m^-3^, hysteresis ratio 0.74 ) with a residual strain (21.6%). Hydrogen bonds densities decreased in the gels with unbalanced ratios of DMAA/AAc, thus, the gell became weak. The maximum stress decreased from 494 KPa (DMAA/AAc= 50/50) to 213 KPa (DMAA/AAc= 75/25). The gel changed from elastic to viscoelastic, that caused bigger hysteresis.


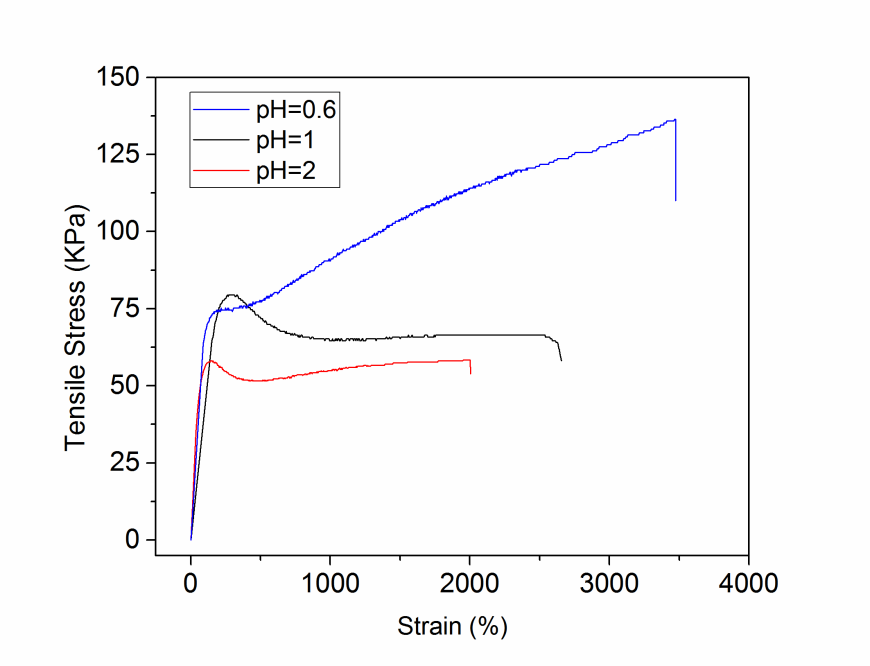


**Figure S5.** Tensile stress-strain curves of hydrogel (R_DMAA/AAc/UPyEA_ = 50/50/0.2) synthesized in different pH solutions.

**
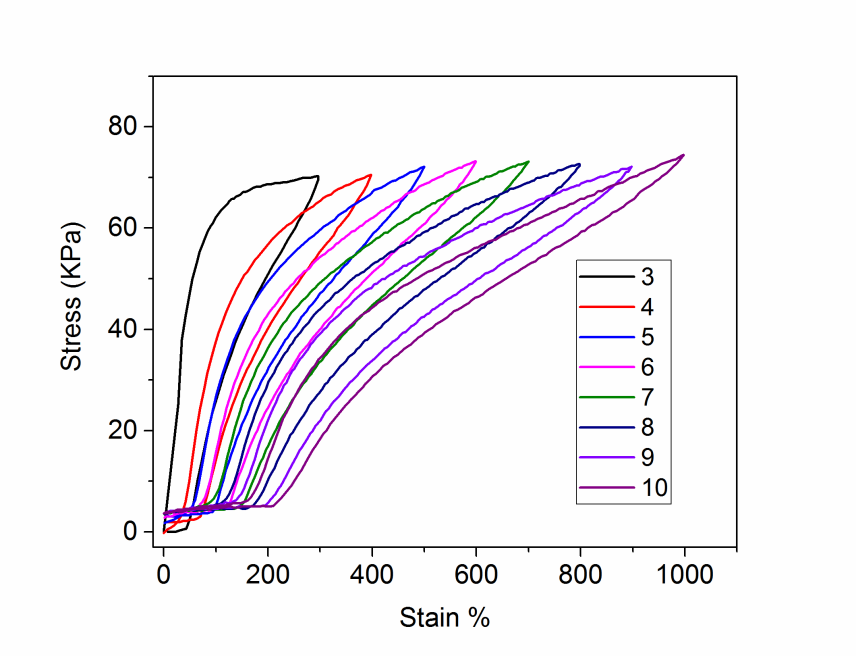
**

**Figure S6.** Samples of the hydrogel (R_DMAA/AAc/UPyEA_ = 50/50/0.2) were subjected to a cycle of loading and unloading of varying maximum stretch (λ = 3-10).
